# The Effects of Hydroalcoholic Extract of Silk Cocoon on Hypothalamic-Pituitary –Gonadal Axis in Streptozotocin-Induced Diabetic Male Rats

**DOI:** 10.1155/2022/7916159

**Published:** 2022-10-19

**Authors:** Salma Ahi, Fatemeh Ebrahimi, Hassan Ali Abedi, Hossein Kargar Jahromi, Safar Zarei

**Affiliations:** ^1^Research Center for Noncommunicable Diseases, Jahrom University of Medical Sciences, Jahrom, Iran; ^2^Student Research Committee, Jahrom University of Medical Sciences, Jahrom, Iran; ^3^Physiology Department, Jahrom University of Medical Sciences, Jahrom, Iran

## Abstract

**Background:**

Diabetes mellitus impairs the reproductive system by damaging the glands and changing their function and hormone secretions. Given the previous studies on medical properties of silk cocoon, the aim of this study was to investigate the effect of the hydroalcoholic extract of silk cocoon on pituitary-gonadal axis hormones and the testis changes in diabetic male rats.

**Methods:**

In this experimental study, 35 male rats were divided into 5 equal groups. Control (C), nontreated diabetic rats (DNT1), and experimental diabetic rats treated (DT1) with a silk cocoon extract at concentrations of 200, 400, and 800 mg/kg for 56 days. Diabetes was induced by an injection of streptozotocin. Blood sampling was performed by the tail and heart after fasting. Body weight, serum levels of glucose, prolactin, leptin, inhibin A, IGF-2, activin A, insulin, LH, testosterone, FSH, and GnRH were measured along with the testis weight and diameter as the outcome of the study. Data were analyzed by SPSS version 20.

**Results:**

Investigation of hormonal factors indicated that all diabetic groups had higher prolactin, inhibin A levels than those in C group and lower leptin, IGF-2, activin A, insulin, LH, testosterone, FSH, and GnRH levels than controls. Silk cocoon treatment significantly decreased prolactin and inhibin in comparison of DNT1 group. While there was a significant increase in leptin, IGF-2, activin A, insulin, LH, testosterone, FSH, and GnRH levels compared with DNT1 (*P* < 0.05). A significant decrease in both the testis weights and diameters was observed in diabetic male rats compared to controls (*P* < 0.05). While silk cocoon treatment improved gonadal weight, the diameter of tunica albuginea, and seminiferous tubules as long as increased in numbers of spermatocytes and Sertoli-Leydig cells. Spermatogonia, spermatocyte, spermatid, spermatozoid, Sertoli cells, and Leydig cell count were significantly lower in the DNT1 group in comparison with the control group, while all groups receiving the highest dose of SC800 mg/kg daily had a higher count of cells than the DNT1 group.

**Conclusion:**

It seems that silk cocoon treatment decreases the effects of diabetes on hypothalamic-pituitary–gonadal axis.

## 1. Background

Diabetes is actually a general term, representing multiple organ involvement and carbohydrate metabolism disorders that is manifested with elevated blood sugar and elevated levels of urine glucose due to insulin secretion deficiency or insulin dysfunction, or both [1]. The prevalence of diabetes mellitus among adults (age 20–79) was about 6.4% in 2010, affecting 285 million people, and is thought to reach 7.7% in 2030 and affect approximately 439 people [2]. Diabetes mellitus causes many disorders, including nephropathy, neuropathy, and retinopathy [3]. There is also evidence that diabetes can affect spermatogenesis and alter testosterone levels and semen volume [4]. Type 1 diabetes is caused by direct damage to the pancreatic beta cells [5]. Its prevalence is approximately 10–15%, and its prevalence gets increased by 3–5% annually [6]. Over 90% of type one diabetes patients are diagnosed before age 30 [7]. As a result, many men of reproductive age are affected by the complications of diabetes [8]. Diabetes affects men's sexual function through alterations in the spermatogenesis process or endocrine changes affecting it, erectile dysfunction and decreased libido, sperm DNA damage, as well as through effects on the hypothalamus and GnRH, by reducing testosterone [9]. In fact, diabetes has altered sperm quality, impaired spermatogenesis, and morphological changes in testicular tissue [9]. In addition, oxidative stress plays an important role in some diabetes-related sexual disorders [10]. Oxidative stress is a condition associated with increased cell damage due to the formation of reactive oxygen species (ROS) in the course of hyperglycemia [11]. Antioxidants reduce cellular damage by delaying the oxidation of other molecules by inhibiting the formation of oxidant chains [12]; larval silkworm is a domestic silk butterfly. One of the silkworms named *Bombyx mori* is one of the first identified silkworm derivatives. It has been widely used in the past few years and has been used in the treatment of various diseases [13]. In traditional medicine, cocoon silk is known as an antidiabetic substance. Existing reports have shown that silk cocoon proteins have strong antioxidant and antidiabetic properties that have been shown to affect hyperglycemia and increase sex hormone levels as well as sperm motility in male mice [14,15]. In the case of silk cocoon, silk is composed of two major polypeptides: sericin and fibroin. Fibroin is a core protein of silk that is composed of 18 different natural amino acids. Fibroin was found to enhance insulin sensitivity and glucose metabolism in 3T3-L1 adipocytes. Sericin is another of two proteins that accounts for 30–30% of the whole cocoon. In the last decade, this protein has gained a great value because of its biological properties, including its ability to absorb moisture and antiapoptotic properties, antioxidant properties, antioxidants, and anti-inflammatory properties. Bacterial antidiabetic noted antidiabetic skin tissue stability [15]. Reducing oxidative stress partially improves serum glucose levels, as well as improving spermatogenesis, results in increased germ cells at different stages [16]. Changes in expression of insulin-like growth factor, androgen receptors, and FSH receptor develop due to diabetes. Serum levels of LH, FSH, and testosterone were significantly reduced. Because of these changes, the function of Leydig cells also is reduced [17]; Researchers have reported that the extract of silk cocoon is effective in improving the oxidative markers of diabetic erythrocytes. Reducing free radicals, simultaneously increasing the activity of antioxidants, and decreasing lipid peroxidation, may reduce the cellular damage. Given that, free radicals are not properly controlled in diabetic patients by oxidation of glucose, nonenzymatic glycation of proteins, and consequently oxidative degradation of glycolytic proteins [18].

It seems that the silk cocoon 's antioxidant properties can reduce the serum glucose level by compensating for the pancreatic function deficits by partially correcting the metabolic disorders caused by increased blood glucose and by increasing insulin secretion. According to the above reports, it can be said that the increase in pituitary-gonadal axis activity, as well as the improvement of testicular tissue damage, are due to the hypoglycemic effects of the silk cocoon.

Due to its role in blood glucose control and direct impact on the levels of the sex hormones and pituitary gland axis, this study aimed to evaluate the silk cocoon effects on male mice fertility.

## 2. Methods

The present study is an experimental study, which was performed on 35 male healthy Wistar rats aged 10 to 12 weeks and weight of 210 ± 10 g. Ethical approval for this study has been received from the Research Ethical Committee of Jahrom University of medical sciences, Iran (Code: IR.JUMS.REC.1398.046). Rats were sourced from Laboratory Animal Breeding Center of Jahrom University of Medical Sciences (Jahrom, Iran). The extraction of silkworm cocoon was done as instructed in a previous study [19].

Animals were maintained at approximately 24–25°C, 45–50% humidity, and 12-hour light/dark cycles. Food and water were supplied in sufficient quantities. All experiments were done at least 10 days after the animals were housed in order to obtain an adaptation to the environment. The adaptation time of the animals was 10 days. Then, 35 male rats were randomly divided into five groups, each containing 7 animals. Of these groups, one group was the control group (C) that received 0.2 cc-distilled water by gavage. The silkworm cocoon was first extracted with ethanol 80%, and then half concentrated after weighed accurately and dissolved in normal saline. The sham or nontreated diabetic group (DNT1) and the other 3 experimental groups were induced diabetes with subcutaneous single injection of 50 mg/kg streptozotocin (STZ) as SC200, SC400, and SC800 received by gavage 200, 400, and 800 mg/kg daily silk cocoon (SC) extract (all dissolved in 0.2 cc-distilled water) for 56 days, respectively. At the end of the study period and after 12 h of fasting, blood tests for glucose level assurance were collected from the tail tip of the rats. The sums of blood glucose of the rats were measured utilizing a glucometer strip (Accu Chek:Germany-made). Then, the rats were weighed and anesthetized with ketamine 80 mg/kg and xylazine 20 mg/kg (Alfasan-Netherland). Blood samples were taken from all animals. Then, blood sampling was done from the left ventricle of the heart using a 5 ml syringe. Blood obtained without an anticoagulant was poured into the test tube and incubated for 12 minutes in an incubator (Memmert UNB 400 Germany) at 37°C. After coagulation, the pipes were place in a centrifuge (Hettich, Germany) for 12 minutes at a speed of 5000 rpm. Serum was separated on the clot section by a sampler (UK, BioPette) and transferred to another test tube, and the obtained serum was stored in a −20°C freezer.

Rats were weighed at 1, 5, 15, and 57 days after study beginning.

The animals were dissected and both testicles were collected and weighed. Testis index was evaluated by the testis weight/body weight. Tissue microscopic specimens for histopathologic evaluation after fixation in 10% formalin solution, dehydrated with alcohol, and then molded in paraffin; finally, tissue passage was conducted. 5-micrometer sections of testes tissue were prepared and examined by hematoxylin-eosin staining. Fifteen slides from each testis (30 slides for each rat) were prepared. For each slide, 10 tubules were assessed. A pathologist examined slides in a blind manner. The average diameter of tunica albuginea and seminiferous tubules, count of spermatogonia, spermatocyte, spermatid, spermatozoid, Sertoli, and Leydig cells (based on the shape of the cell and nucleus and the position of placement in the tubule) was observed by a Nikon microscope in the eyepiece 10× and objective lens 40× and measured by the Dino Capture Soft Imaging System (Japan).

A micrometer measured the thicknesses of averagely fifty points of tunica albuginea from each section [20].

The diameter of seminiferous tubules was measured using the method of Singh Sudamani et al. Twenty-five tubules were randomly selected and the mean tubule diameter was calculated by a calibrated micrometer connected to the microscope eyepiece and using the following formula: mean diameter = √ *l* × *b* × magnification, where *l* is the length and *b* is the breadth of the tubule [21].

Data were analyzed by SPSS software version 20. Data were analyzed by one-way ANOVA and Duncan post hoc tests. The results were reported in figures drawn by excel. The level of significance was considered 0.05.

## 3. Results

**Figure 1:**
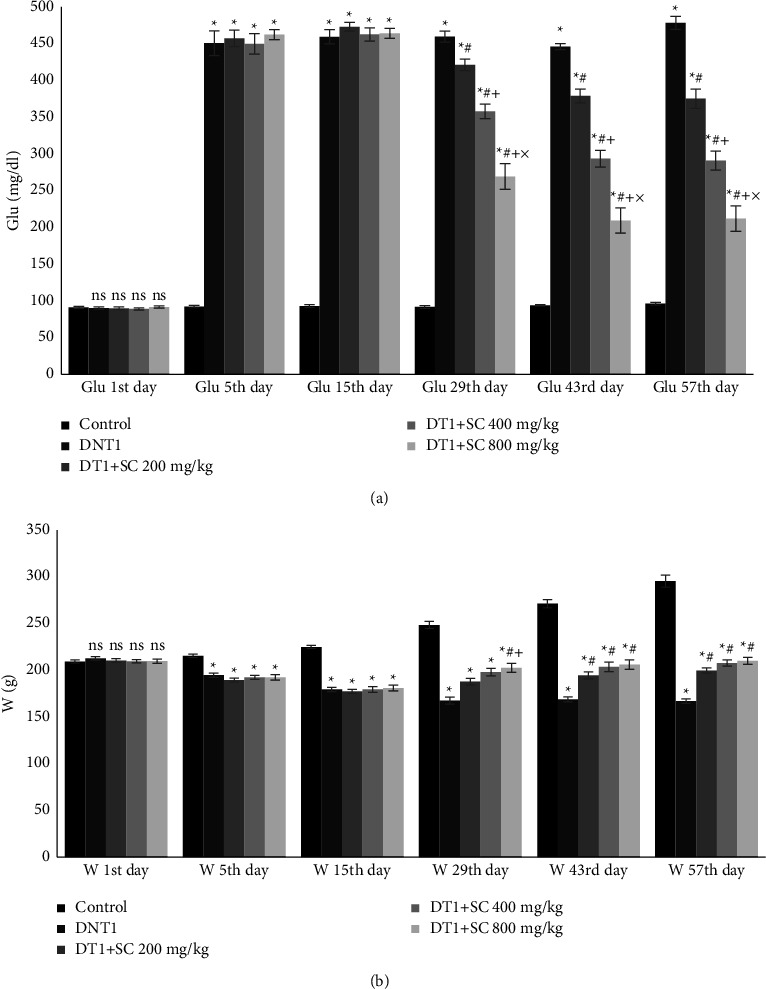
Blood glucose and body weight of animals during the study. (a) Mean glucose level and (b) mean body weight. Significant differences at *p* value < 0.05 are shown as not having a common character. Values are shown as mean ± SEM. ns: nonsignificant between groups, star sign (^*∗*^): difference with control, hashtag sign (#): difference with DNT1, plus sign (+): difference with DT1 + SC200 mg, multiplication sign (×): difference with DT1 + SC400 mg.

As shown in Figure 1, the results of study revealed significantly increased glucose level of all study groups (DNT1, DT1 + SC200, DT1 + SC400, DT1 + SC800) in comparison to the control group, after STZ treatment after 5,15, and 57 days (*P* < 0.001). There was not any significant difference between groups treated with silk cocoon (DT1 + SC200, DT1 + SC400, DT1 + SC800) and DiabetT1 groups, at 5 (*P*=0.994, *P*=1, *P*=0.949) and 15 days (*P*=0.671, *P*=0.998, *P*=0.99) after diabetes induction. While on day 57, D + SC200, D1 + SC400, and DT1 + SC800 groups had significantly lower glucose level than the DNT1 group (*P* < 0.001). Glucose levels of all study groups after STZ treatment on 29 and 43 days are provided in supplementary table file (S1).

Body weight on the first day in all groups (DNT1 + SC200, DT1 + SC400, DT1 + SC800) did not show a significant difference from the control group (*P*=0.746, *P*=0.996, *P*=1, *P*=1). Body weight of diabetic animals (DNT1, DT1 + SC200, DT1 + SC400, DT1 + SC800) was significantly lower than the control group at 5, 15, and 57 days (*P* < 0.001). While on day 57, silk cocoon group (DT1 + SC200, DT1 + SC400, DT1 + SC800) had a significantly higher weight than DNT1 (*P* < 0.001). Body weights of animals on 29th and 43th day of treatment are provided in supplementary table file (S1).

**Figure 2:**
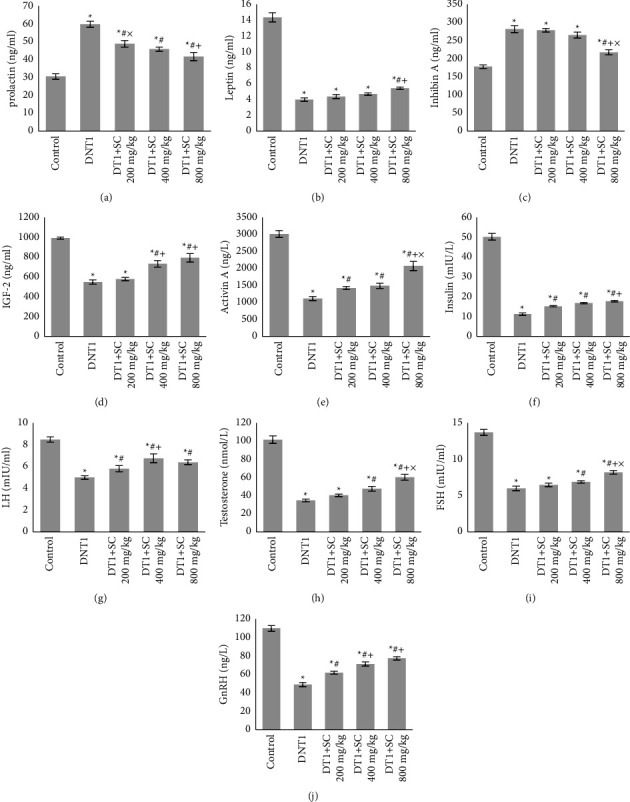
Blood prolactin, leptin, inhibin A IGF-2, activin A insulin, LH, testosterone, FSH, and GnRH levels. (a) Mean prolactin level, (b) mean leptin, (b) inhibin A, (c) IGF-2, (d) activin A, (e) insulin, (f) LH, (h): testosterone, (i) FSH, and (j) GnRH. Significant differences at *p* value <0.05 are shown as not having a common character. Values are shown as mean ± SEM. ns: nonsignificant between groups, hashtag sign (#): difference with DNT1, plus sign (+): difference with DT1 + SC200 mg, multiplication sign (×): difference with DT1 + SC400 mg.

As shown in Figure 2, the investigation of hormonal factors indicated that all study groups (DNT1, DT1 + SC200, DT1 + SC400, DT1 + SC800) had higher prolactin (*P* < 0.001) and inhibin A level than the control group (*P* < 0.001, *P* < 0.001, *P* < 0.001, *P*=0.003). Lower levels of leptin (*P* < 0.001), IGF-2 (*P* < 0.001), activin A (*P* < 0.001), insulin (*P* < 0.001), LH (*P* < 0.001)), testosterone (*P* < 0.001), FSH (*P* < 0.001), and GnRH were observed in these groups than controls (*P* < 0.001).

Silk cocoon treatments (DNT1 + SC200, DT1 + SC400, DT1 + SC800) significantly decreased the prolactin levels in comparison of the DNT1 group (*P*=0.001, *P* < 0.001, *P* < 0.001). Inhibin A showed a significant decrease (*P* < 0.001) and leptin showed a significant increase (*P*=0.019) only in the DT1 + SC800 group with the DNT1 group (*P*=0.019).

Silk cocoon treatments (DNT1 + SC200, DT1 + SC400, DT1 + SC800) significantly increased insulin (*P*=0.018, *P* < 0.001, *P* < 0.001) and GnRH levels (*P*=0.003, *P* < 0.001, *P* < 0.001) in comparison with the DNT1 group.

Silk cocoon treatments (DT1 + SC400, DT1 + SC800) significantly increased IGF-2 (*P* < 0.001), activin A (*P*=0.038, *P* < 0.001), LH (*P*=0.001, *P*=0.009), testosterone (*P*=0.022, *P* < 0.000), and FSH (*P*=0.02, *P* < 0.000) levels in comparison with the DNT1 group (*P* < 0.05).

**Figure 3:**
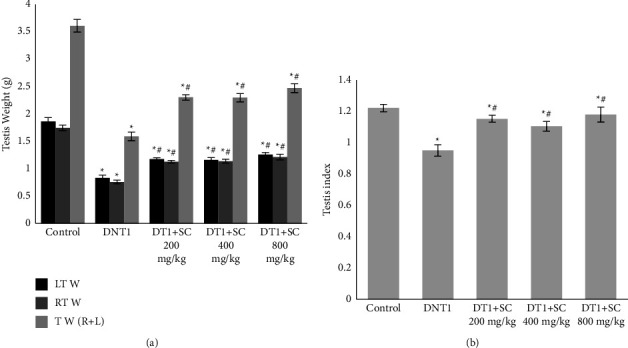
Testis weight and testis index of animals. (a) mean testis weight, (b): mean testis index. Significant differences at *p* value <0.05 are shown as not having a common character. Values are shown as Mean ± SEM. star sign (^*∗*^): difference with control, hashtag sign (#): difference with DNT1, plus sign (+): difference with DT1 + SC200 mg, multiplication sign (×): difference with DT1 + SC400 mg.

Evaluating the testis weight as outcomes of our study revealed a significant decrease in diabetic groups (DNT1, DT1 + SC200, DT1 + SC400, DT1 + SC800) in comparison to control. Left testis weight (*P* < 0.001), right testis weight (*P* < 0.001), and both testis weight (*P* < 0.001) in diabetic groups were decreased in comparison to control. While all SC treated groups (DT1 + SC200, DT1 + SC400, DT1 + SC800) had higher left testis weight (*P* < 0.001), right testis weight (*P* < 0.001), and both testis weight (*P* < 0.001) than DNT1 group (Figure 3).

Evaluating the testis index showed a significant decrease in diabetic groups (DNT1, DT1 + SC400) in comparison to control (*P* < 0.001, *P*=0.012). While SC treated groups (DT1 + SC200, DT1 + SC400, DT1 + SC800) had higher testis index (*P*=0.001, *P*=0.019, *P* < 0.001) than DNT1 group.

**Figure 4:**
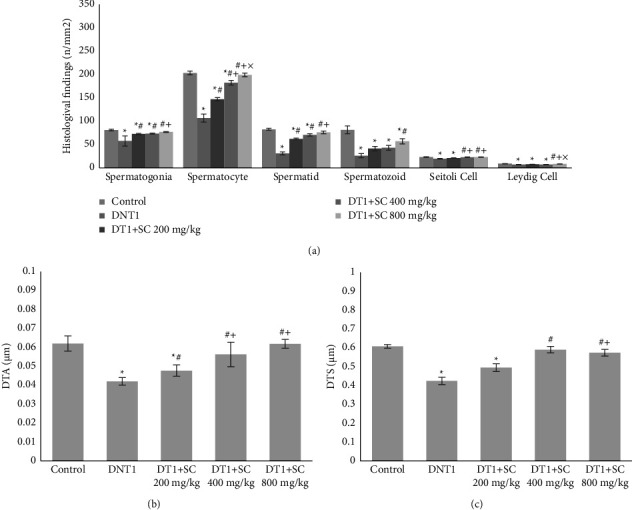
(a) Histological findings of cells count: spermatogonia, spermatocyte, spermatid, spermatozoid, sertoli cell, leydig cell count in mm^2^. (b) Diameter of tunica albuginea. (c) Diameter of seminiferous tubule. Significant differences at *p* value <0.05 are shown as not having common character. Values are shown as mean ± SEM. star sign (^*∗*^): difference with control, hashtag sign (#): difference with DNT1, plus sign (+): difference with DT1 + SC200 mg, multiplication sign (×): difference with DT1 + SC400 mg.

**Figure 5:**
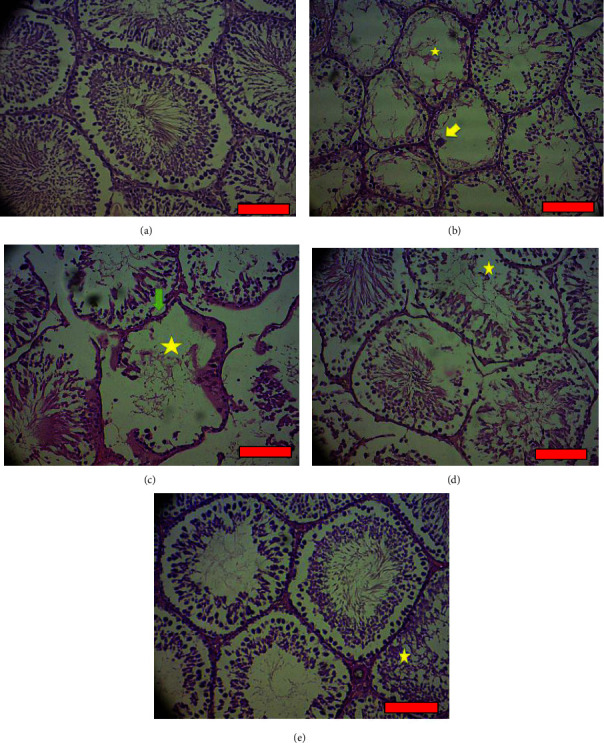
Results of histopathological changes in testicular tissues in different groups: yellow arrow = presence of tissue macrophage cell in seminiferous tubules. Star = destruction of seminiferous tubules and disruption of cellular order. Green arrow = folding of the base tube of the seminiferous tubules. Red line = scale bar (100 *μ*m). (a) Control group: H&E (100% magnification). (b) T1DM group: H&E (100% magnification). (c) Diabetes and silk group 200 mg: H&E (100% magnification). (d) Diabetes and silk group 400 mg: H&E (100% magnification). (e) Diabetes and silk group 800 mg: H&E (100% magnification).

As shown in Figure 4, spermatogonia, spermatocyte, spermatid, spermatozoid, Sertoli Cell, and Leydig Cell count were significantly lower in DNT1 group in comparison to the control group (*P* < 0.001). While all groups receiving SC800, had a higher count of cells than DNT1 group) *P* < 0.001, *P* < 0.001, *P* < 0.001, *P*=0.002, *P* < 0.001, *P* < 0.001). Histological H&E samples are shown in Figure 5.

Regarding silk cocoon effects on study variables with different doses, the most effects on decreased in serum PRL level, serum inhibin A and mean blood glucose levels after 15 days as long as increased in serum levels of IGF2, insulin, testosterone, FSH, and GnRH were observed with dose 800 mg/kg of silk cocoon. Furthermore, an increase in the number of spermatogonia and spermatocytes, and Leydig cells also increased in diameter of tunica albuginea, seminiferous tubules, and total body weight of rats were observed with 800 mg/kg silk cocoon. 400 mg/kg silk cocoon is the most effective dose in increasing serum level of LH, the number of Sertoli cells, and testes diameter.

### 3.1. Results of Histopathological Changes in Testicular Tissues in Different Groups

No tissue changes were observed in the control group (Figure 5(a)). In the diabetic group, very severe destruction of seminiferous tubules was observed with disruption of cellular order inside the tubule and the presence of macrophage cells along (Figure 5(b)). However, these changes happened with less severity in the diabetic group receiving silk cocoon extract (200 mg) (Figure 5(c)). In the diabetic group receiving silk cocoon extract (400 mg), the changes were again less than that seen in the group receiving silk cocoon extract (200 mg) (Figure 5(d)). In the diabetic group receiving silk cocoon extract with a concentration of 800 mg, all mentioned changes happened with moderate intensity (Figure 5(e)). All figures are provided in supplementary figure file (S2).

## 4. Discussion

The hypothalamic-pituitary-gonadal axis contains multidisciplinary hormones that bind to the neuroendocrine system, and their function is essential for regulating fertility. Disruption or depletion of either of these hormones results in impaired fertility [22]. Hormones involved in this axis, such as leptin, GnRH, and insulin secretion by stimulating hypothalamic neurons, which directly increase the secretion of GnRH in the hypothalamus [23]. In diabetes, the stimulating effects of GnRH hormone are reduced, leading to a decrease in the secretion of other sex hormones and ultimately erectile dysfunction and the number, motility, and quality of sperm reduction [4].

In our study, streptozotocin treatment significantly increased glucose, prolactin, and inhibin A levels of all study groups. Streptozotocin-induced diabetes effect on fertility is well established in animal research studies [24].

In a study of streptozotocin-induced diabetic rats, serum levels of testosterone were significantly decreased in diabetic rats compared to healthy rats, suggesting that diabetes had negative effects on reproductive system function and structure. Male mice decreased testosterone production, secretion, and decreased spermatogenesis [25]. Research study indicates the important role of reactive oxygen species in causing testicular tissue damage in diabetic rats. Oxidative stress resulting from an imbalance between free radical production and antioxidant defense capacity is strongly associated with diabetes and its complications; DNA damage caused by free radicals can speed up the process of sex cell apoptosis and reduce the number of germ cells. Diabetes also causes testicular tissue damage and impaired spermatogenesis through atrophy of spermatozoa, reduced spermatogenic tubule diameter, and reduced spermatogenic cell line. During diabetes, there are changes in the expression of insulin-like growth factor, androgen receptors, and FSH receptor in the testis. On the other hand, the levels of LH, FSH, and testosterone are significantly reduced in the blood serum, and because of these changes, the function of Leydig cells is reduced [26].

A study was carried out to investigate the effect of diabetes and silk cocoon on gonadotropin and gonadal hormone changes in male rats. It was reported that diabetes reduces body weight and testicular weight loss in comparison to healthy mice and the serum levels of LH, FSH, testosterone, 17-estradiol and progesterone decreased in diabetic rats compared to healthy rats [27].

Studies have shown that diabetes reduces testosterone production and secretion by reducing the number of Leydig cells in diabetic rats [28]. Diabetes has been reported to decrease testicular tissue perfusion and the oxidative stress induced by diabetes affects gonadotropin hormone secretion and testicular function and may decrease function by causing multiple damages to testicular tissue, especially Leydig and Sertoli cells [29].

The results of a study showed that diabetes leads to a significant decrease in serum testosterone levels. Diabetes-induced oxidative stress can play an important and key role in Leydig cell function, decrease its activity, and lead to a decrease in testosterone synthesis and secretion [30].

Research has shown that diabetes causes oxidative stress in the pituitary gland cells and as a gonadotropin-secreting gland; it can affect the activity of the pituitary-gonadal axis, sex hormones, and fertility rates. The activity of LH and FSH depends on both the serum levels of these two hormones and the number of receptors in the testis. Diabetes and its oxidative stress are associated with increased levels of free cell radicals and the effect of LH, FSH, and serum levels of these hormones [17].

Diabetes has been reported to decrease spermatogenesis by reducing the secretion of gonadotropins and by directly affecting Leydig and Sertoli cells involved in the production and transfer of testosterone. According to the results of this study, it can be concluded that a decrease in the pituitary-gonadal axis activity and a decrease in serum sex hormone concentration in male rats are a consequence of diabetes mellitus, which is consistent with recent studies [31].

It is likely that the antioxidant compounds of the plant may increase the cellular function the pituitary-gonadal axis and enhance the activity of the hypothalamic axis. In addition, the secretion of testicular Leydig cells is increased by decreasing oxidative stress.

Diabetes can decrease sperm count due to impaired spermatogenesis with a mechanism dependent on FSH. Numerous studies have shown the therapeutic effects of medicinal plants in preventing sperm reduction in diabetes, which also indicate different genera of the Suschian family. This is the reality [32]. One of them is an aqueous extract of garlic, which has therapeutic and preventive effects on testicular tissue damage in diabetic rats and prevents the reduction of spermatogenesis. Carrot seed extract can also increase testosterone and spermatogenesis in testicular tissue in male rats. Oxidative stress, lipid peroxidation, and altered membrane.

Property causes germ cell death at different stages of spermatogenesis and consequently decreases sperm count. On the other hand, antioxidants control and suppress the production of free radicals [33]. Among the available antioxidants, plant sources have attracted the attention of many researchers today. Research has shown that antioxidants can be effective in treating male infertility by reducing the damage caused by free radicals, enhancing the blood-testicular barrier, and protecting and repairing sperm DNA [34].

## 5. Conclusion

In conclusion, dose-dependent administration of extract of silk cocoon plant increases the level of sex hormone secretion in diabetic rats by affecting the activity of pituitary-gonadal axis. In addition, with its protective effect on the testis weight, dose-dependent administration of aqueous extract of silk cocoon plant can improve testicular tissue injuries in type 1 diabetic rats.

## Data Availability

There are no additional data. All data generated or analyzed during this study are included in this published article.

## Abbreviations

STZ: Streptozotocin
DMT1: Diabetes mellitus type 1
SC: Silk cocoon
H&E: Hematoxylin and Eosin.

## Supplementary Materials

Supplementary Materials Variables of all study groups after STZ treatment on 29, 43 days are provided in supplementary table (S1). Histopathological changes in testicular tissue of different groups are provided as S2.
