# Supplementary material for: The Effects of Hydroalcoholic Extract of Silk Cocoon on Hypothalamic-Pituitary –Gonadal Axis in Streptozotocin-Induced Diabetic Male Rats
Source: Autoimmune Dis. 2022 Oct 19;2022:7916159. doi: 10.1155/2022/7916159 (PMC9605846; doi:10.1155/2022/7916159)
Supplement: Supplementary Materials — Variables of all study groups after STZ treatment on 29, 43 days are provided in supplementary table (S1). Histopathological changes in testicular tissue of different groups are provided as S2. [file 7916159.f1.zip › S1.docx]

S1: supplementary table file

ONEWAY FBS29 FBS43 W29 W43 BY GROUP

/STATISTICS DESCRIPTIVES

/MISSING ANALYSIS

/POSTHOC=BTUKEY DUNCAN LSD ALPHA(0.05).

**Oneway**

| **Notes** | | |
| --- | --- | --- |
| Output Created | | 03-JUL-2022 23:50:34 |
| Comments | |  |
| Input | Active Dataset | DataSet0 |
|  | Filter | <none> |
|  | Weight | <none> |
|  | Split File | <none> |
|  | N of Rows in Working Data File | 40 |
| Missing Value Handling | Definition of Missing | User-defined missing values are treated as missing. |
|  | Cases Used | Statistics for each analysis are based on cases with no missing data for any variable in the analysis. |
| Syntax | | ONEWAY FBS29 FBS43 W29 W43 BY GROUP  /STATISTICS DESCRIPTIVES  /MISSING ANALYSIS  /POSTHOC=BTUKEY DUNCAN LSD ALPHA(0.05). |
| Resources | Processor Time | 00:00:00.20 |
|  | Elapsed Time | 00:00:00.30 |

[DataSet0]

| **Descriptives** | | | | | | |
| --- | --- | --- | --- | --- | --- | --- |
|  | | N | Mean | Std. Deviation | Std. Error | 95% Confidence Interval for Mean |
|  |  |  |  |  |  | Lower Bound |
| FBS29 | CONTROL | 8 | 91.5000 | 5.12696 | 1.81265 | 87.2138 |
|  | DT1 | 8 | 459.6250 | 20.73601 | 7.33129 | 442.2893 |
|  | DT1+SC200 | 8 | 421.1250 | 22.29950 | 7.88407 | 402.4821 |
|  | DT1+SC400 | 8 | 357.8750 | 28.02773 | 9.90930 | 334.4432 |
|  | DT1+SC800 | 8 | 269.1250 | 49.36580 | 17.45345 | 227.8542 |
|  | Total | 40 | 319.8500 | 135.57883 | 21.43689 | 276.4898 |
| FBS43 | CONTROL | 8 | 93.7500 | 3.19598 | 1.12995 | 91.0781 |
|  | DT1 | 8 | 446.0000 | 11.66190 | 4.12311 | 436.2504 |
|  | DT1+SC200 | 8 | 378.8750 | 26.48686 | 9.36452 | 356.7314 |
|  | DT1+SC400 | 8 | 293.5000 | 32.18695 | 11.37981 | 266.5910 |
|  | DT1+SC800 | 8 | 209.2500 | 48.62612 | 17.19193 | 168.5975 |
|  | Total | 40 | 284.2750 | 128.76932 | 20.36022 | 243.0926 |
| W29 | CONTROL | 8 | 248.2500 | 10.96423 | 3.87644 | 239.0837 |
|  | DT1 | 8 | 167.3750 | 10.68961 | 3.77935 | 158.4383 |
|  | DT1+SC200 | 8 | 187.6250 | 10.22514 | 3.61513 | 179.0766 |
|  | DT1+SC400 | 8 | 197.8750 | 11.55654 | 4.08585 | 188.2135 |
|  | DT1+SC800 | 8 | 202.3750 | 13.54292 | 4.78815 | 191.0528 |
|  | Total | 40 | 200.7000 | 29.09683 | 4.60061 | 191.3944 |
| W43 | CONTROL | 8 | 271.1250 | 12.32231 | 4.35659 | 260.8233 |
|  | DT1 | 8 | 168.7500 | 7.90569 | 2.79508 | 162.1407 |
|  | DT1+SC200 | 8 | 194.5000 | 10.62342 | 3.75595 | 185.6186 |
|  | DT1+SC400 | 8 | 203.5000 | 14.24279 | 5.03559 | 191.5927 |
|  | DT1+SC800 | 8 | 205.8750 | 14.60369 | 5.16319 | 193.6660 |
|  | Total | 40 | 208.7500 | 36.17603 | 5.71993 | 197.1803 |

| **Descriptives** | | | | |
| --- | --- | --- | --- | --- |
|  | | 95% Confidence Interval for Mean | Minimum | Maximum |
|  |  | Upper Bound |  |  |
| FBS29 | CONTROL | 95.7862 | 85.00 | 99.00 |
|  | DT1 | 476.9607 | 427.00 | 486.00 |
|  | DT1+SC200 | 439.7679 | 389.00 | 455.00 |
|  | DT1+SC400 | 381.3068 | 315.00 | 392.00 |
|  | DT1+SC800 | 310.3958 | 216.00 | 337.00 |
|  | Total | 363.2102 | 85.00 | 486.00 |
| FBS43 | CONTROL | 96.4219 | 89.00 | 98.00 |
|  | DT1 | 455.7496 | 427.00 | 466.00 |
|  | DT1+SC200 | 401.0186 | 327.00 | 411.00 |
|  | DT1+SC400 | 320.4090 | 243.00 | 335.00 |
|  | DT1+SC800 | 249.9025 | 154.00 | 286.00 |
|  | Total | 325.4574 | 89.00 | 466.00 |
| W29 | CONTROL | 257.4163 | 235.00 | 264.00 |
|  | DT1 | 176.3117 | 151.00 | 186.00 |
|  | DT1+SC200 | 196.1734 | 175.00 | 201.00 |
|  | DT1+SC400 | 207.5365 | 179.00 | 211.00 |
|  | DT1+SC800 | 213.6972 | 177.00 | 216.00 |
|  | Total | 210.0056 | 151.00 | 264.00 |
| W43 | CONTROL | 281.4267 | 250.00 | 287.00 |
|  | DT1 | 175.3593 | 159.00 | 180.00 |
|  | DT1+SC200 | 203.3814 | 182.00 | 208.00 |
|  | DT1+SC400 | 215.4073 | 183.00 | 221.00 |
|  | DT1+SC800 | 218.0840 | 183.00 | 223.00 |
|  | Total | 220.3197 | 159.00 | 287.00 |

| **ANOVA** | | | | | | |
| --- | --- | --- | --- | --- | --- | --- |
|  | | Sum of Squares | df | Mean Square | F | Sig. |
| FBS29 | Between Groups | 687650.600 | 4 | 171912.650 | 205.831 | .000 |
|  | Within Groups | 29232.500 | 35 | 835.214 |  |  |
|  | Total | 716883.100 | 39 |  |  |  |
| FBS43 | Between Groups | 616942.100 | 4 | 154235.525 | 181.528 | .000 |
|  | Within Groups | 29737.875 | 35 | 849.654 |  |  |
|  | Total | 646679.975 | 39 |  |  |  |
| W29 | Between Groups | 28426.400 | 4 | 7106.600 | 54.166 | .000 |
|  | Within Groups | 4592.000 | 35 | 131.200 |  |  |
|  | Total | 33018.400 | 39 |  |  |  |
| W43 | Between Groups | 45836.250 | 4 | 11459.063 | 77.080 | .000 |
|  | Within Groups | 5203.250 | 35 | 148.664 |  |  |
|  | Total | 51039.500 | 39 |  |  |  |

**Post Hoc Tests**

| **Multiple Comparisons** | | | | | | | | |
| --- | --- | --- | --- | --- | --- | --- | --- | --- |
| Dependent Variable | | (I) GROUP | (J) GROUP | Mean Difference (I-J) | Std. Error | Sig. | 95% Confidence Interval | |
|  |  |  |  |  |  |  | Lower Bound | Upper Bound |
| FBS29 | LSD | CONTROL | DT1 | -368.12500^*^ | 14.45004 | .000 | -397.4601 | -338.7899 |
|  |  |  | DT1+SC200 | -329.62500^*^ | 14.45004 | .000 | -358.9601 | -300.2899 |
|  |  |  | DT1+SC400 | -266.37500^*^ | 14.45004 | .000 | -295.7101 | -237.0399 |
|  |  |  | DT1+SC800 | -177.62500^*^ | 14.45004 | .000 | -206.9601 | -148.2899 |
|  |  | DT1 | CONTROL | 368.12500^*^ | 14.45004 | .000 | 338.7899 | 397.4601 |
|  |  |  | DT1+SC200 | 38.50000^*^ | 14.45004 | .012 | 9.1649 | 67.8351 |
|  |  |  | DT1+SC400 | 101.75000^*^ | 14.45004 | .000 | 72.4149 | 131.0851 |
|  |  |  | DT1+SC800 | 190.50000^*^ | 14.45004 | .000 | 161.1649 | 219.8351 |
|  |  | DT1+SC200 | CONTROL | 329.62500^*^ | 14.45004 | .000 | 300.2899 | 358.9601 |
|  |  |  | DT1 | -38.50000^*^ | 14.45004 | .012 | -67.8351 | -9.1649 |
|  |  |  | DT1+SC400 | 63.25000^*^ | 14.45004 | .000 | 33.9149 | 92.5851 |
|  |  |  | DT1+SC800 | 152.00000^*^ | 14.45004 | .000 | 122.6649 | 181.3351 |
|  |  | DT1+SC400 | CONTROL | 266.37500^*^ | 14.45004 | .000 | 237.0399 | 295.7101 |
|  |  |  | DT1 | -101.75000^*^ | 14.45004 | .000 | -131.0851 | -72.4149 |
|  |  |  | DT1+SC200 | -63.25000^*^ | 14.45004 | .000 | -92.5851 | -33.9149 |
|  |  |  | DT1+SC800 | 88.75000^*^ | 14.45004 | .000 | 59.4149 | 118.0851 |
|  |  | DT1+SC800 | CONTROL | 177.62500^*^ | 14.45004 | .000 | 148.2899 | 206.9601 |
|  |  |  | DT1 | -190.50000^*^ | 14.45004 | .000 | -219.8351 | -161.1649 |
|  |  |  | DT1+SC200 | -152.00000^*^ | 14.45004 | .000 | -181.3351 | -122.6649 |
|  |  |  | DT1+SC400 | -88.75000^*^ | 14.45004 | .000 | -118.0851 | -59.4149 |
| FBS43 | LSD | CONTROL | DT1 | -352.25000^*^ | 14.57441 | .000 | -381.8376 | -322.6624 |
|  |  |  | DT1+SC200 | -285.12500^*^ | 14.57441 | .000 | -314.7126 | -255.5374 |
|  |  |  | DT1+SC400 | -199.75000^*^ | 14.57441 | .000 | -229.3376 | -170.1624 |
|  |  |  | DT1+SC800 | -115.50000^*^ | 14.57441 | .000 | -145.0876 | -85.9124 |
|  |  | DT1 | CONTROL | 352.25000^*^ | 14.57441 | .000 | 322.6624 | 381.8376 |
|  |  |  | DT1+SC200 | 67.12500^*^ | 14.57441 | .000 | 37.5374 | 96.7126 |
|  |  |  | DT1+SC400 | 152.50000^*^ | 14.57441 | .000 | 122.9124 | 182.0876 |
|  |  |  | DT1+SC800 | 236.75000^*^ | 14.57441 | .000 | 207.1624 | 266.3376 |
|  |  | DT1+SC200 | CONTROL | 285.12500^*^ | 14.57441 | .000 | 255.5374 | 314.7126 |
|  |  |  | DT1 | -67.12500^*^ | 14.57441 | .000 | -96.7126 | -37.5374 |
|  |  |  | DT1+SC400 | 85.37500^*^ | 14.57441 | .000 | 55.7874 | 114.9626 |
|  |  |  | DT1+SC800 | 169.62500^*^ | 14.57441 | .000 | 140.0374 | 199.2126 |
|  |  | DT1+SC400 | CONTROL | 199.75000^*^ | 14.57441 | .000 | 170.1624 | 229.3376 |
|  |  |  | DT1 | -152.50000^*^ | 14.57441 | .000 | -182.0876 | -122.9124 |
|  |  |  | DT1+SC200 | -85.37500^*^ | 14.57441 | .000 | -114.9626 | -55.7874 |
|  |  |  | DT1+SC800 | 84.25000^*^ | 14.57441 | .000 | 54.6624 | 113.8376 |
|  |  | DT1+SC800 | CONTROL | 115.50000^*^ | 14.57441 | .000 | 85.9124 | 145.0876 |
|  |  |  | DT1 | -236.75000^*^ | 14.57441 | .000 | -266.3376 | -207.1624 |
|  |  |  | DT1+SC200 | -169.62500^*^ | 14.57441 | .000 | -199.2126 | -140.0374 |
|  |  |  | DT1+SC400 | -84.25000^*^ | 14.57441 | .000 | -113.8376 | -54.6624 |
| W29 | LSD | CONTROL | DT1 | 80.87500^*^ | 5.72713 | .000 | 69.2483 | 92.5017 |
|  |  |  | DT1+SC200 | 60.62500^*^ | 5.72713 | .000 | 48.9983 | 72.2517 |
|  |  |  | DT1+SC400 | 50.37500^*^ | 5.72713 | .000 | 38.7483 | 62.0017 |
|  |  |  | DT1+SC800 | 45.87500^*^ | 5.72713 | .000 | 34.2483 | 57.5017 |
|  |  | DT1 | CONTROL | -80.87500^*^ | 5.72713 | .000 | -92.5017 | -69.2483 |
|  |  |  | DT1+SC200 | -20.25000^*^ | 5.72713 | .001 | -31.8767 | -8.6233 |
|  |  |  | DT1+SC400 | -30.50000^*^ | 5.72713 | .000 | -42.1267 | -18.8733 |
|  |  |  | DT1+SC800 | -35.00000^*^ | 5.72713 | .000 | -46.6267 | -23.3733 |
|  |  | DT1+SC200 | CONTROL | -60.62500^*^ | 5.72713 | .000 | -72.2517 | -48.9983 |
|  |  |  | DT1 | 20.25000^*^ | 5.72713 | .001 | 8.6233 | 31.8767 |
|  |  |  | DT1+SC400 | -10.25000 | 5.72713 | .082 | -21.8767 | 1.3767 |
|  |  |  | DT1+SC800 | -14.75000^*^ | 5.72713 | .014 | -26.3767 | -3.1233 |
|  |  | DT1+SC400 | CONTROL | -50.37500^*^ | 5.72713 | .000 | -62.0017 | -38.7483 |
|  |  |  | DT1 | 30.50000^*^ | 5.72713 | .000 | 18.8733 | 42.1267 |
|  |  |  | DT1+SC200 | 10.25000 | 5.72713 | .082 | -1.3767 | 21.8767 |
|  |  |  | DT1+SC800 | -4.50000 | 5.72713 | .437 | -16.1267 | 7.1267 |
|  |  | DT1+SC800 | CONTROL | -45.87500^*^ | 5.72713 | .000 | -57.5017 | -34.2483 |
|  |  |  | DT1 | 35.00000^*^ | 5.72713 | .000 | 23.3733 | 46.6267 |
|  |  |  | DT1+SC200 | 14.75000^*^ | 5.72713 | .014 | 3.1233 | 26.3767 |
|  |  |  | DT1+SC400 | 4.50000 | 5.72713 | .437 | -7.1267 | 16.1267 |
| W43 | LSD | CONTROL | DT1 | 102.37500^*^ | 6.09640 | .000 | 89.9987 | 114.7513 |
|  |  |  | DT1+SC200 | 76.62500^*^ | 6.09640 | .000 | 64.2487 | 89.0013 |
|  |  |  | DT1+SC400 | 67.62500^*^ | 6.09640 | .000 | 55.2487 | 80.0013 |
|  |  |  | DT1+SC800 | 65.25000^*^ | 6.09640 | .000 | 52.8737 | 77.6263 |
|  |  | DT1 | CONTROL | -102.37500^*^ | 6.09640 | .000 | -114.7513 | -89.9987 |
|  |  |  | DT1+SC200 | -25.75000^*^ | 6.09640 | .000 | -38.1263 | -13.3737 |
|  |  |  | DT1+SC400 | -34.75000^*^ | 6.09640 | .000 | -47.1263 | -22.3737 |
|  |  |  | DT1+SC800 | -37.12500^*^ | 6.09640 | .000 | -49.5013 | -24.7487 |
|  |  | DT1+SC200 | CONTROL | -76.62500^*^ | 6.09640 | .000 | -89.0013 | -64.2487 |
|  |  |  | DT1 | 25.75000^*^ | 6.09640 | .000 | 13.3737 | 38.1263 |
|  |  |  | DT1+SC400 | -9.00000 | 6.09640 | .149 | -21.3763 | 3.3763 |
|  |  |  | DT1+SC800 | -11.37500 | 6.09640 | .070 | -23.7513 | 1.0013 |
|  |  | DT1+SC400 | CONTROL | -67.62500^*^ | 6.09640 | .000 | -80.0013 | -55.2487 |
|  |  |  | DT1 | 34.75000^*^ | 6.09640 | .000 | 22.3737 | 47.1263 |
|  |  |  | DT1+SC200 | 9.00000 | 6.09640 | .149 | -3.3763 | 21.3763 |
|  |  |  | DT1+SC800 | -2.37500 | 6.09640 | .699 | -14.7513 | 10.0013 |
|  |  | DT1+SC800 | CONTROL | -65.25000^*^ | 6.09640 | .000 | -77.6263 | -52.8737 |
|  |  |  | DT1 | 37.12500^*^ | 6.09640 | .000 | 24.7487 | 49.5013 |
|  |  |  | DT1+SC200 | 11.37500 | 6.09640 | .070 | -1.0013 | 23.7513 |
|  |  |  | DT1+SC400 | 2.37500 | 6.09640 | .699 | -10.0013 | 14.7513 |

| *. The mean difference is significant at the 0.05 level. |
| --- |

**Homogeneous Subsets**

| **FBS29** | | | | | | | |
| --- | --- | --- | --- | --- | --- | --- | --- |
|  | GROUP | N | Subset for alpha = 0.05 | | | | |
|  |  |  | 1 | 2 | 3 | 4 | 5 |
| Tukey B^a^ | CONTROL | 8 | 91.5000 |  |  |  |  |
|  | DT1+SC800 | 8 |  | 269.1250 |  |  |  |
|  | DT1+SC400 | 8 |  |  | 357.8750 |  |  |
|  | DT1+SC200 | 8 |  |  |  | 421.1250 |  |
|  | DT1 | 8 |  |  |  |  | 459.6250 |
| Duncan^a^ | CONTROL | 8 | 91.5000 |  |  |  |  |
|  | DT1+SC800 | 8 |  | 269.1250 |  |  |  |
|  | DT1+SC400 | 8 |  |  | 357.8750 |  |  |
|  | DT1+SC200 | 8 |  |  |  | 421.1250 |  |
|  | DT1 | 8 |  |  |  |  | 459.6250 |
|  | Sig. |  | 1.000 | 1.000 | 1.000 | 1.000 | 1.000 |

| Means for groups in homogeneous subsets are displayed. |
| --- |
| a. Uses Harmonic Mean Sample Size = 8.000. |

| **FBS43** | | | | | | | |
| --- | --- | --- | --- | --- | --- | --- | --- |
|  | GROUP | N | Subset for alpha = 0.05 | | | | |
|  |  |  | 1 | 2 | 3 | 4 | 5 |
| Tukey B^a^ | CONTROL | 8 | 93.7500 |  |  |  |  |
|  | DT1+SC800 | 8 |  | 209.2500 |  |  |  |
|  | DT1+SC400 | 8 |  |  | 293.5000 |  |  |
|  | DT1+SC200 | 8 |  |  |  | 378.8750 |  |
|  | DT1 | 8 |  |  |  |  | 446.0000 |
| Duncan^a^ | CONTROL | 8 | 93.7500 |  |  |  |  |
|  | DT1+SC800 | 8 |  | 209.2500 |  |  |  |
|  | DT1+SC400 | 8 |  |  | 293.5000 |  |  |
|  | DT1+SC200 | 8 |  |  |  | 378.8750 |  |
|  | DT1 | 8 |  |  |  |  | 446.0000 |
|  | Sig. |  | 1.000 | 1.000 | 1.000 | 1.000 | 1.000 |

| Means for groups in homogeneous subsets are displayed. |
| --- |
| a. Uses Harmonic Mean Sample Size = 8.000. |

| **W29** | | | | | | |
| --- | --- | --- | --- | --- | --- | --- |
|  | GROUP | N | Subset for alpha = 0.05 | | | |
|  |  |  | 1 | 2 | 3 | 4 |
| Tukey B^a^ | DT1 | 8 | 167.3750 |  |  |  |
|  | DT1+SC200 | 8 |  | 187.6250 |  |  |
|  | DT1+SC400 | 8 |  | 197.8750 |  |  |
|  | DT1+SC800 | 8 |  | 202.3750 |  |  |
|  | CONTROL | 8 |  |  | 248.2500 |  |
| Duncan^a^ | DT1 | 8 | 167.3750 |  |  |  |
|  | DT1+SC200 | 8 |  | 187.6250 |  |  |
|  | DT1+SC400 | 8 |  | 197.8750 | 197.8750 |  |
|  | DT1+SC800 | 8 |  |  | 202.3750 |  |
|  | CONTROL | 8 |  |  |  | 248.2500 |
|  | Sig. |  | 1.000 | .082 | .437 | 1.000 |

| Means for groups in homogeneous subsets are displayed. |
| --- |
| a. Uses Harmonic Mean Sample Size = 8.000. |

| **W43** | | | | | |
| --- | --- | --- | --- | --- | --- |
|  | GROUP | N | Subset for alpha = 0.05 | | |
|  |  |  | 1 | 2 | 3 |
| Tukey B^a^ | DT1 | 8 | 168.7500 |  |  |
|  | DT1+SC200 | 8 |  | 194.5000 |  |
|  | DT1+SC400 | 8 |  | 203.5000 |  |
|  | DT1+SC800 | 8 |  | 205.8750 |  |
|  | CONTROL | 8 |  |  | 271.1250 |
| Duncan^a^ | DT1 | 8 | 168.7500 |  |  |
|  | DT1+SC200 | 8 |  | 194.5000 |  |
|  | DT1+SC400 | 8 |  | 203.5000 |  |
|  | DT1+SC800 | 8 |  | 205.8750 |  |
|  | CONTROL | 8 |  |  | 271.1250 |
|  | Sig. |  | 1.000 | .086 | 1.000 |

| Means for groups in homogeneous subsets are displayed. |
| --- |
| a. Uses Harmonic Mean Sample Size = 8.000. |
