# Supplementary material for: The Effects of Hydroalcoholic Extract of Silk Cocoon on Hypothalamic-Pituitary –Gonadal Axis in Streptozotocin-Induced Diabetic Male Rats
Source: Autoimmune Dis. 2022 Oct 19;2022:7916159. doi: 10.1155/2022/7916159 (PMC9605846; doi:10.1155/2022/7916159)
Supplement: Supplementary Materials — Variables of all study groups after STZ treatment on 29, 43 days are provided in supplementary table (S1). Histopathological changes in testicular tissue of different groups are provided as S2. [file 7916159.f1.zip › S2.doc]

**Results of histo-pathological changes in testicular tissue in different groups:**

No tissue changes were observed in the control group (Figures a to d). In the diabetic group, very severe destruction of seminiferous tubules was observed with disruption of cellular order inside the tubule and the presence of macrophage cells along with increased Tunica Albuginea thickness (Figures e to h). However these changes happened with less severity in the diabetic group receiving silk cocoon extract (200 mg) (Figures i to n). In the diabetic group receiving silk cocoon extract (400 mg) the changes were again less than that of seen in group receiving silk cocoon extract (200 mg) (Figures o to r).

In the diabetic group receiving silk cocoon extract with a concentration of 800 mg all mentioned changes happened with a moderate intensity (Figures s to v).

|  |  |
| --- | --- |
| a) Control group: H&E (40% magnification): No tissue changes are observed. | b) Control group: H&E (100% magnification): No tissue changes are observed. |
|  |  |
| c) Control group: (H&E 100%) magnification No tissue changes are observed. | d) Control group: (H&E 400%) magnification No tissue changes are observed. |

|  |  |
| --- | --- |
| e) T1DM Group: H&E (40% magnification): Red Arrow = Increased Tonic Albogina, Star = Destruction of Semenifer Tubes and Disruption of Cellular Order. | f) T1DM group: H&E (100% magnification): Yellow Arrow = Presence of Tissue Macrophage Cell in Semenfer Tools , Star = destruction of seminiferous tubules and disruption of cellular order |
|  |  |
| g) type 1 diabetes group: H&E (400% magnification) yellow arrow = presence of tissue macrophage cells in seminiferous tubules, star = destruction of seminiferous tubules and disruption of cellular order | h) type 1 diabetes group: H&E (400% magnification): green Arrow=folding of the base membrane of the seminiferous tubule, star = destruction of the seminal vesicle and disruption of cellular order |

|  |  |
| --- | --- |
| i) Diabetes and Silk Group 200 mg: H&E (40% magnification): Green Arrow = Folding of the base membrane of the seminal vesicle, Star = Destruction of the seminal vesicle and disruption of cellular order | j) Diabetes and silk group 200 mg: H&E (100% magnification): green arrow = Folding of the base tube of the seminal vesicle, star = Destruction of the esophageal tube and confusion of the cellular order |
|  |  |
| l) Diabetes and silk group 200 mg: H&E (100% magnification): the green arrow = Folding of the primary membrane of the esophageal tube, star = destruction of the seminal vesicle Cellular Order | n) Diabetes and Silk Group 200 mg: H&E (40% magnification): Red Arrow = Increases Tonic Albogina Thickness, Star = Destruction of Semnifer Tubers and Cellular Disorder |

|  |  |
| --- | --- |
| o) Diabetes and Silk Group 400 mg: H&E Enlargement 40 × Green Flash = Folding of the base membrane of the seminal vesicle, Star = Destruction of the seminal vesicle and disruption of cellular order | p) Diabetes and silk group 400 mg: H&E Enlargement 40% of the green arrow = Folding of the base membrane of the seminal vesicle, star = destruction of the seminal vesicle and disruption of cellular order |
|  |  |
| q) Diabetes and silk group 400 mg: H&E 100% magnification of the star = destruction of the seminal vesicle and disruption of cellular order | r) Diabetes and silk group 400 ml Warm: H&E 40% magnification red flash = increase the thickness of Tonica Albogina |

|  |  |
| --- | --- |
| s) Diabetes and silk group 800 mg: H&E 40% star magnification = destruction of seminiferous tubules and disruption of cellular order | t) Diabetes and silk group 800 mg: H&E magnification 40% green flash = folding of the base membrane of semen transfer tubes, star = Destruction of the seminiferous tubule and disruption of cellular order |
|  |  |
| u) Diabetes and silk group 800 mg: H&E 100% star magnification = Destruction of the seminiferous tubule and disruption of cellular order | v) Diabetes and silk group 800 mg: H&E magnification 40% red flash = increase Tonica Albogina thickness |
